# Metabolic Profiling of Cochlear Organoids Identifies α‐Ketoglutarate and NAD^+^ as Limiting Factors for Hair Cell Reprogramming

**DOI:** 10.1002/advs.202308032

**Published:** 2024-07-11

**Authors:** Qing Liu, Linqing Zhang, Zhen Chen, Yihan He, Yuhang Huang, Cui Qiu, Chengwen Zhu, Danxia Zhou, Zhenji Gan, Xia Gao, Guoqiang Wan

**Affiliations:** ^1^ State Key Laboratory of Pharmaceutical Biotechnology MOE Key Laboratory of Model Animal for Disease Study and Jiangsu Provincial Key Medical Discipline (Laboratory) Department of Otolaryngology Head and Neck Surgery Affiliated Drum Tower Hospital of Medical School Model Animal Research Center of Medical School Nanjing University Nanjing 210032 China; ^2^ State Key Laboratory of Pharmaceutical Biotechnology MOE Key Laboratory of Model Animal for Disease Study and Jiangsu Key Laboratory of Molecular Medicine Model Animal Research Center of Medical School Nanjing University Nanjing 210032 China; ^3^ Research Institute of Otolaryngology No. 321 Zhongshan Road Nanjing 210008 China

**Keywords:** α‐ketoglutarate, cochlear organoids, hair cells, NAD^+^, reprogramming

## Abstract

Cochlear hair cells are the sensory cells responsible for transduction of acoustic signals. In mammals, damaged hair cells do not regenerate, resulting in permanent hearing loss. Reprogramming of the surrounding supporting cells to functional hair cells represent a novel strategy to hearing restoration. However, cellular processes governing the efficient and functional hair cell reprogramming are not completely understood. Employing the mouse cochlear organoid system, detailed metabolomic characterizations of the expanding and differentiating organoids are performed. It is found that hair cell differentiation is associated with increased mitochondrial electron transport chain (ETC) activity and reactive oxidative species generation. Transcriptome and metabolome analyses indicate reduced expression of oxidoreductases and tricyclic acid (TCA) cycle metabolites. The metabolic decoupling between ETC and TCA cycle limits the availability of the key metabolic cofactors, α‐ketoglutarate (α‐KG) and nicotinamide adenine dinucleotide (NAD^+^). Reduced expression of NAD^+^ in cochlear supporting cells by PGC1α deficiency further impairs hair cell reprogramming, while supplementation of α‐KG and NAD^+^ promotes hair cell reprogramming both in vitro and in vivo. These findings reveal metabolic rewiring as a central cellular process during hair cell differentiation, and highlight the insufficiency of key metabolites as a metabolic barrier for efficient hair cell reprogramming.

## Graphical abstract

Regeneration of cochlear hair cells holds great promise for hearing restoration. Cochlear organoids have emerged as valuable system for high throughput analyses of mammalian hair cell development. This study suggests that metabolic decoupling is an underlying barrier for efficient hair cell regeneration, and points to the roles of key metabolites such as *α*‐ketoglutarate and nicotinamide adenine dinucleotidein promoting hair cell differentiation.

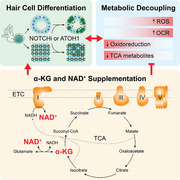

## 1 Introduction

Degeneration of the cochlear sensory hair cells is the major cause of sensorineural hearing loss.^[^ ^1^, ^2^ ^]^ Unlike birds and amphibians, hair cells in mammals do not spontaneously regenerate, posing a great challenge to hearing recovery.^[^ ^3^, ^4^, ^5^, ^6^ ^]^ Attempts have been made to convert the supporting cells, which surround the sensory hair cells, into hair cell‐like cells.^[^ ^7^, ^8^, ^9^ ^]^ Key signaling pathways (FGF, BMP, Wnt, Notch, Hippo, and Shh) and transcription factors (Atoh1, Pou4f3, Gfi1, Six1, Gata3, p27Kip1, and Myc) have been exploited for induced renewal of supporting cell and reprogramming into hair cells.^[^ ^7^, ^8^ ^]^ Despite these efforts, the efficiency of hair cell reprogramming and the function of the induced hair cells remain limited.^[^ ^10^ ^]^

In addition to modulations of signaling pathways and transcription factors, efficient and functional cell reprogramming requires the removal of additional roadblocks, such as epigenetic regulation, immunomodulation, remodeling of extracellular environments, and reprogramming of metabolic states.^[^ ^11^, ^12^, ^13^ ^]^ Recent studies indicate that cellular metabolism, apart from its crucial role in energy production, also regulates the fates of both stem cells and differentiated cells through mechanisms such as cellular redox balance, transcriptional regulation, and histone epigenetic modifications.^[^ ^14^, ^15^ ^]^ Importantly, during cell transdifferentiation and tissue regeneration in neurons, muscles, and liver, effective control of cell fate can be achieved by regulating cellular oxygen consumption, controlling energy metabolism, and maintaining critical levels of key metabolites.^[^ ^13^, ^16^, ^17^ ^]^ However, whether and how metabolic rewiring contributes to hair cell reprogramming is unclear.

Recent developments in the inner ear and cochlear organoids have provided unprecedented opportunities for high throughput screening,^[^ ^18^ ^]^ regenerative pathway identification,^[^ ^19^, ^20^, ^21^, ^22^ ^]^ otic lineage analyses,^[^ ^23^, ^24^, ^25^ ^]^ and disease modeling studies,^[^ ^26^, ^27^ ^]^ owning to their resemblance to inner ear developmental patterns^[^ ^28^, ^29^, ^30^, ^31^, ^32^ ^]^ and capabilities to expand in large quantities in vitro.^[^ ^18^, ^31^ ^]^ In this study, we performed both transcriptomic and metabolomic profiling of the expanding and differentiated cochlear organoids derived from neonatal mouse cochlear sensory epithelia. We found that hair cell reprogramming is accompanied by increased oxygen consumption rates and production of reactive oxygen species, related to reduced levels of tricarboxylic acid cycle metabolites and oxidoreductase activities. Interestingly, replenishment of α‐ketoglutarate or nicotinamide adenine dinucleotide significantly promotes hair cell reprogramming both in vitro and in vivo, thus providing new therapeutic targets for hair cell regeneration and hearing restoration.

## 2 Results

### 2.1 Increased Oxygen Consumption Rate and Reactive Oxygen Species Generation During Hair Cell Differentiation

To understand the biochemical processes underlying hair cell differentiation, we first performed bulk transcriptomic analyses on mouse cochlear organoids at either the expansion (DIV10) or differentiation (DIV20) stages (Figure S1A,B, Supporting Information). As expected, gene ontology (GO) analysis showed that processes related to hair cell differentiation and function were upregulated (Figure S1C, Supporting Information). Multiple hair cell markers (*Otof*, *Tmc1*, *Gfi1*, etc.) were highly upregulated in the differentiated cochlear organoids (Figure S1D,E, Supporting Information), consistent with our previous report.^[^ ^18^ ^]^ Not surprisingly, differentiated cochlear organoids showed downregulated processes in cell proliferation and DNA replication (Figure S2A,B, Supporting Information). Interestingly, genes involved in oxidoreductive activities were significantly downregulated (Figure S2B,C).

**Figure 1:**
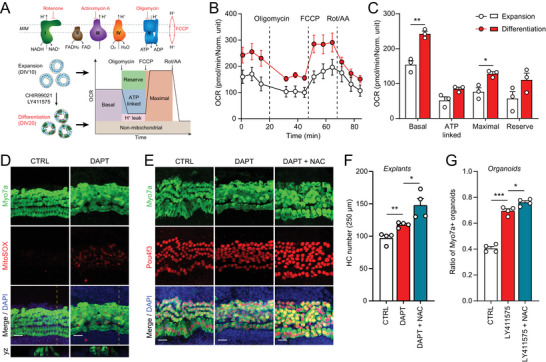
Increased oxygen consumption rate and ROS generation during hair cell differentiation. A) Schematic of oxygen consumption tests using cochlear organoids. MIM, mitochondrial inner membrane. B) Oxygen consumption rate (OCR) of cochlear organoids during expansion (DIV10) and differentiation (DIV20) stages. Basal OCR was first measured, followed by administration of oligomycin (5 µm), FCCP (5 µm), or rotenone/antimycin A (Rot/AA; 2 µM). C) Basal, ATP‐linked, maximal, and reserve OCR were calculated as shown in (A). Error bars represent mean ± SEM. n = 8 biological replicates with each replicate containing 30–60 organoids. ^*^ *P* < 0.05 and ^**^ *P* < 0.01 by unpaired *t*‐test. D) DAPT‐induced ROS generation in cochlear explants during hair cell differentiation. Mitochondrial ROS was labeled with mitoSOX. (Scale bars: 20 µm) E,F) Confocal images and hair cell counts show NAC treatment increased the efficiency of hair cell differentiation. (Scale bars: 20 µm) Error bars represent mean ± SEM. n = 4 cochlear explants at each condition. ^*^ *P* < 0.05 and ^**^ *P* < 0.01 by unpaired *t‐*test. G) NAC treatment increased the efficiency of hair cell differentiation in cochlear organoids. Error bars represent mean ± SD. n = 4 biological replicates with each replicate containing 150–300 organoids. ^*^ *P* < 0.05 and ^***^ *P* < 0.001 by unpaired *t‐*test.

The electron transport chain (ETC), part of cellular respiration and oxidative phosphorylation (OXPHOS), is the major process regulating cellular oxidoreduction.^[^ ^33^ ^]^ We therefore examined the ETC activity by measuring oxygen consumption rates (OCRs) of both expanding and differentiated organoids^[^ ^34^ ^]^ (**Figure** **1**A). Both basal and maximal OCRs were significantly higher in the differentiated organoids, compared to the expanding ones (Figures 1B,C), suggestive of increased ETC activity during hair cell differentiation. Increased ETC activity is accompanied by the production of reactive oxygen species (ROS), which are important for redox‐related pathophysiological processes.^[^ ^35^ ^]^ Indeed, when we induced hair cell differentiation in cochlear explants using the *γ*‐secretase inhibitor DAPT, robust mitochondrial ROS production was observed (Figure 1D). The ROS scavenger NAC promoted hair cell differentiation in both cochlear explants (Figure 1E,F) and organoids (Figure 1G).

To address whether increased oxygen consumption is causal to the hair cell differentiation, we treated the differentiating cochlear organoids or explants in both normoxia (21% O_2_) and hypoxia (5% O_2_) conditions. Hypoxia significantly inhibited hair cell differentiation in both cochlear organoids (Figure S3A,B, Supporting Information) and explants (Figure S3C,D, Supporting Information), indicating that the normal oxygen level is essential for efficient hair cell differentiation. Furthermore, to explore if increased ATP levels were involved in efficient hair cell differentiation, we co‐treated the cochlear explants with various concentrations of creatine, which is shown to increase cellular ATP levels by enhancing the *de novo* ATP synthesis.^[^ ^36^, ^37^, ^38^ ^]^ Interestingly, creatine only had mild effects on the efficiency of hair cell differentiation (Figure S3E,F, Supporting Information), suggesting that while ATP is critical for the myriad of cellular functions, it is not a limiting factor for hair cell differentiation.

Together, our results indicate that hair cell differentiation is associated with reduced oxidoreductive activities, increased oxygen consumption, and ROS generation that may reduce the efficiency of hair cell reprogramming from the supporting cells.

### 2.2 Reduced TCA Cycle Metabolites During Hair Cell Differentiation

Alterations in the ETC activity and mitochondrial respiration (Figure 1A–C) prompted us to examine the metabolic changes during hair cell differentiation, a task that may only be achieved by large quantities of cochlear organoids. Non‐targeted metabolomic analyses were then performed using cochlear organoids (2000–3000 per replicate) at both expansion and differentiation stages (Figure S4 and Dataset S1, Supporting Information). PCA analyses demonstrated differential profiles of metabolites in replicates from expanding and differentiated organoids (Figure S4B, Supporting Information), while both heatmap (Figure S4A, Supporting Information) and volcano (Figure S4C, Supporting Information) plots showed differentially regulated metabolites during hair cell differentiation.

**Figure 2:**
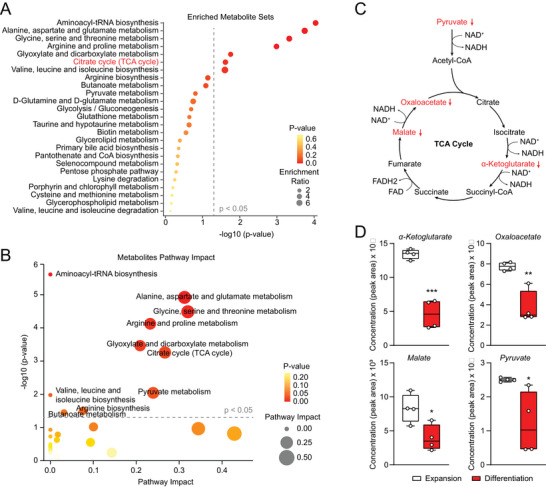
Reduced TCA cycle metabolites during hair cell differentiation. A,B) Enriched metabolite sets and Metabolic pathway impact of differentially regulated metabolites during hair cell differentiation. Non‐targeted metabolomic analyses were performed using cochlear organoids at expansion (DIV10) and differentiation (DIV20) stages. C) Oxidative phosphorylation pathway showing the downregulated metabolites pyruvate and TCA intermediates α‐ketoglutarate, malate and oxaloacetate. D) Concentrations of TCA cycle metabolites during hair cell differentiation in cochlear organoids. n = 4 biological replicates with each replicate containing 2000–3000 organoids. ^*^ *P* < 0.05, ^**^ *P* < 0.01, and ^***^ *P* < 0.001 by unpaired *t*‐test.

Analyses of the enriched metabolite sets and pathway impact suggested that multiple metabolic processes were regulated during hair cell differentiation, including amino acid metabolism and aminoacyl‐tRNA biosynthesis (**Figure** **2**A,B). Interestingly, the tricarboxylic acid (TCA) cycle or citrate cycle was among the significantly regulated metabolic pathways (Figure 2A–C). Pyruvate, the metabolic precursor of the TCA cycle, and multiple TCA cycle intermediates (i.e., oxaloacetate, malate, α‐ketoglutarate), showed reduced amounts in differentiated organoids (Figure 2C,D).

A number of free amino acids, such as alanine, glycine, threonine, tryptophan, proline, and valine, etc., may be metabolized as precursors for TCA cycle intermediates (Figure S5A, Supporting Information). Similarly, these free amino acids were also downregulated during hair cell differentiation in cochlear organoids (Figure S5B, Supporting Information), consistent with reduced abundance of the TCA cycle intermediates (Figure 2C,D). KEGG analysis of the RNA‐seq results shows significant upregulation of protein digestion and absorption pathway during organoid differentiation (Figure S5C, Supporting Information). Correspondingly, multiple dipeptides, generated by protein digestion, accumulated during hair cell differentiation (Figure S5D, Supporting Information). These data indicate that both TCA cycle intermediates and the precursor‐free amino acids are consumed during hair cell differentiation, which activates a cellular feedback mechanism by upregulation of protein absorption and digestion.

We next examined the expression of key TCA cycle enzymes to explore if they correlated with the abundance of TCA cycle intermediates during hair cell differentiation (Figure S6A, Supporting Information). Surprisingly, *Mpc1*, *Mpc2*, *Aco2*, *Idh1*, *Idh2*, *Dlst*, and *Sdha* were upregulated in the differentiated organoids, compared to the expanding ones (Figure S6B, Supporting Information). Upregulation of Sdha protein expression was also validated by western blot analysis (Figure S6C,D, Supporting Information). The TCA cycle enzymes were also enriched in hair cells of the mature cochlea (Figure S6E, Supporting Information), consistent with their upregulation during hair cell differentiation in organoids. These results suggest that hair cells exhibit a high demand for oxygen consumption and TCA cycle metabolism, while reduced TCA cycle intermediates may impede efficient hair cell differentiation.

### 2.3 α‐Ketoglutarate (α‐KG) Promotes Hair Cell Differentiation

**Figure 3:**
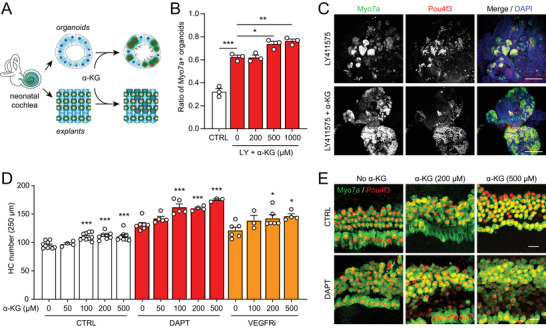
α‐Ketoglutarate (α‐KG) promotes hair cell differentiation. A) Schematic of α‐KG treatment in cochlear organoids and explants. B) Co‐treatment of α‐KG with LY411575 (LY) increased the ratio of Myo7a+ cochlear organoids. Error bars represent mean ± SD. n = 3 biological replicates with each replicate containing 150–300 organoids. ^*^ *P* < 0.05, ^**^ *P* < 0.01 and ^***^ *P* < 0.001 by one‐way ANOVA. C) Confocal images of cochlear organoids differentiated with and without co‐treatment of α‐KG. (Scale bars: 100 µm) D) α‐KG alone or co‐treatment with DAPT or Regorafenib (VEGFRi) promoted hair cell differentiation in cochlear explants. Error bars represent mean ± SEM. n = 3–10 cochlear explants at each condition. ^*^ *P* < 0.05 and ^***^ *P* < 0.001 by one‐way ANOVA, comparing to no α‐KG within each group. E) Confocal images of cochlear explants treated with various concentrations of α‐KG alone or with DAPT. (Scale bars: 20 µm).

α‐Ketoglutarate (α‐KG), also known as 2‐oxoglutarate, is a multi‐faceted metabolite involved in the pleiotropic metabolic and regulatory pathways in virtually all organisms.^[^ ^39^ ^]^ Notably, α‐KG also plays important roles in cell fate determination and lineage specification, such as timing and differentiation of the germ cells, skeletal stem cells, and pancreatic cells.^[^ ^40^, ^41^, ^42^, ^43^ ^]^ To determine the role of α‐KG in hair cell differentiation, we co‐treated the differentiating cochlear organoids and explants with *γ*‐secretase inhibitors and various concentrations of α‐KG (**Figure** **3**A). The efficiency of hair cell differentiation in cochlear organoids was significantly improved by co‐treatment of α‐KG in a dose‐dependent manner (Figure 3B,C). Similarly, co‐treatment of α‐KG also enhanced hair cell differentiation in culture explants, with effective concentrations ranging from 100 to 500 µm (Figure 3D,E). The synergistic effect of α‐KG was observed with both DAPT and VEGFRi, which is a small molecule that enhances hair cell differentiation independent of Notch signaling.^[^ ^18^ ^]^ Interestingly, α‐KG also promoted hair cell differentiation under control conditions (CHIR99021 alone). These data suggest that α‐KG may be essential to fulfill a generic metabolic demand for efficient hair cell differentiation.

### 2.4 NAD^+^ is Increased by α‐KG and Promotes Hair Cell Differentiation

**Figure 4:**
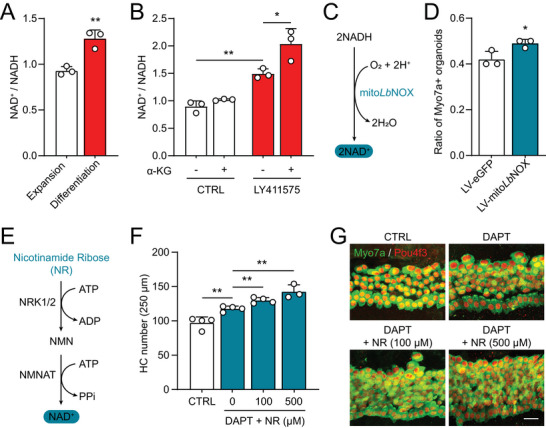
NAD^+^ is increased by α‐KG and promotes hair cell differentiation. A) Increased NAD^+^/NADH ratio during hair cell differentiation. Cochlear organoids at expansion (DIV10) or differentiation (DIV20) stages were subjected to NAD^+^/NADH assay. Error bars represent mean ± SD. n = 3 biological replicates with each replicate containing 200–300 organoids. ^**^ *P* < 0.01 by unpaired *t*‐test. B) α‐KG further increased NAD^+^ and NADH ratios during hair cell differentiation. Cochlear organoids were differentiated in the presence or absence of LY411575 and α‐KG, and subjected to NAD^+^/NADH assay at DIV20. A,B) Error bars represent mean ± SD. n = 3 biological replicates with each replicate containing 200–300 organoids. *P < 0.05 and **P < 0.01 by unpaired *t* test. C) Conversion of NADH to NAD^+^ by mito*Lb*NOX. D) Lentivirus‐mediated overexpression of mito*Lb*NOX promoted hair cell differentiation in cochlear organoids. Cochlear organoids were infected with control (LV‐eGFP) or mito*Lb*NOX‐eGFP lentivirus at DIV2, differentiated with DAPT, and analyzed at DIV20. The number of Myo7a/eGFP double‐positive organoids was normalized to the number of total eGFP+ organoids. Error bars represent mean ± SD. n = 3 biological replicates with each replicate containing 150–300 organoids. ^*^ *P* < 0.05 by unpaired *t* test. E) Generation of NAD^+^ from the precursor nicotinamide ribose (NR). F) NR co‐treatment with DAPT promoted hair cell differentiation in cochlear explants. Error bars represent mean ± SEM. n = 3–4 cochlear explants at each condition. ^**^ *P* < 0.01 by one‐way ANOVA. G) Confocal images of cochlear explants co‐treated with DAPT and various concentrations of NR. (Scale bar: 20 µm).

As a cofactor, NAD^+^ plays pivotal roles in multiple metabolic pathways, including TCA, ETC, fatty acid oxidation, and alcohol metabolisms.^[^ ^44^ ^]^ Because hair cell differentiation was accompanied by the metabolic decoupling of decreased TCA cycle metabolites and increased ETC activity (Figure 1), we next investigated if NAD^+^, which participates in both metabolic pathways, may be regulated by hair cell differentiation and by α‐KG. The ratio of NAD^+^ to NADH, which measures the relative abundance of the oxidized NAD^+^, was significantly increased during hair cell differentiation in cochlear organoids (**Figure** **4**A). Interestingly, NAD^+^/NADH ratio can be further increased by co‐treatment of α‐KG (Figure 4B), suggesting that the efficiency of hair cell differentiation correlates with the NAD^+^/NADH ratio. *Lb*NOX is a water‐forming NADH oxidase from *Lactobacillus brevis*. LbNOX couples the oxidation of NADH to NAD^+^ with the reduction of oxygen to water.^[^ ^45^ ^]^ Mito*Lb*NOX is tagged with a mitochondrial localization signal that has improved activity.^[^ ^46^ ^]^ To directly examine the role of NAD^+^ in hair cell differentiation, we constructed a lentiviral vector expressing mito*Lb*NOX that converts mitochondrial NADH to NAD^+^ (Figure 4C).^[^ ^45^, ^46^ ^]^ Indeed, lentiviral expression of mito*Lb*NOX significantly increased the efficiency of hair cell differentiation in cochlear organoids (Figure 4D). We also co‐treated the cochlear explants with nicotinamide ribose (NR), a precursor of NAD^+^ (Figure 4E). Similarly, NR promoted hair cell differentiation in a dose‐dependent manner in cochlear explants (Figure 4F,G). These results suggest that increased NAD^+^ level not only accompanies but also promotes hair cell differentiation.

### 2.5 PGC1α Knockout Reduces NAD^+^ and Impairs Hair Cell Differentiation

**Figure 5:**
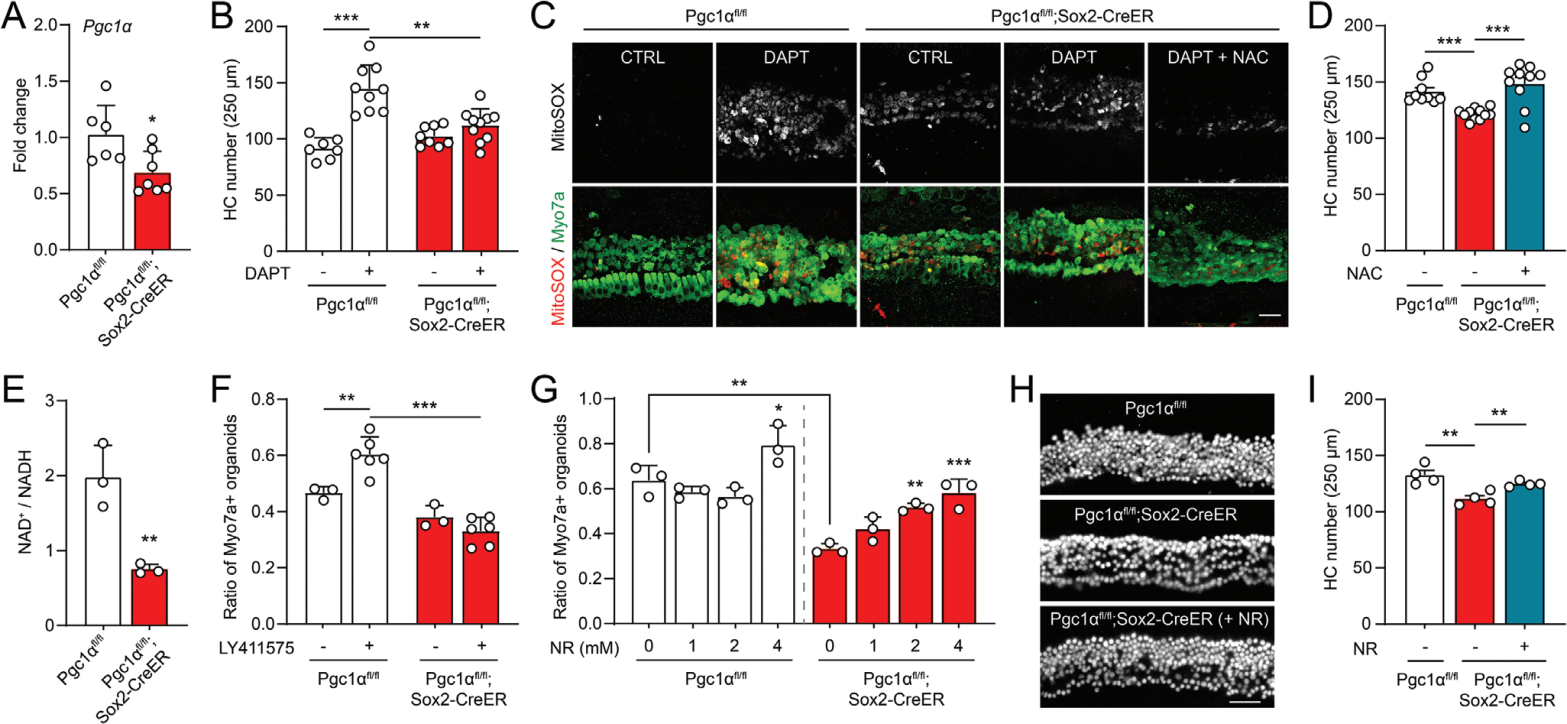
PGC1α knockout reduced NAD^+^ and impairs hair cell differentiation. A) RT‐qPCR validation of PGC1α (Ppargc1a) knockout in cochlear organoids derived from PGC1α^fl/fl^;Sox2‐CreER mice. PGC1α knockout was induced by 4‐OH tamoxifen treatment starting from DIV2. PGC1α^fl/fl^ organoids were used as controls. Error bars represent mean ± SD. n = 6–7 biological replicates with each replicate containing150–300 organoids. ^*^ *P* < 0.05 by unpaired *t‐*test. B) PGC1α knockout impaired hair cell differentiation in cochlear explants. Error bars represent mean ± SEM. n = 7–10 cochlear explants at each condition. ^**^ *P* < 0.01 and ^***^ *P* < 0.001 by unpaired *t* test. C) PGC1α knockout induced ROS generation in cochlear explants regardless of DAPT treatment, and NAC scavenged ROS production. Mitochondrial ROS was labeled with mitoSOX. (Scale bar: 20 µm) D) Effect of PGC1α knockout on hair cell differentiation was restored by co‐treatment of NAC. Error bars represent mean ± SEM. n = 9–11 cochlear explants at each condition. ^***^ *P* < 0.001 by unpaired *t*‐test. E) Reduced NAD^+^/NADH ratio in PGC1α knockout organoids. Error bars represent mean ± SD. n = 3 biological replicates with each replicate containing 200–300 organoids. ^**^ *P* < 0.01 by unpaired *t*‐test. F) PGC1α knockout impaired hair cell differentiation in cochlear organoids. Error bars represent mean ± SD. n = 3–6 biological replicates with each replicate containing 150–300 organoids. ^**^ *P* < 0.01 and ^***^ *P* < 0.001 by unpaired *t* test. G) The effect of PGC1α knockout on hair cell differentiation was restored by co‐treatment of NR. Error bars represent mean ± SD. n = 3 biological replicates with each replicate containing 150–300 organoids. ^*^ *P* < 0.05, ^**^ *P* < 0.01, and ^***^ *P* < 0.001 by one‐way ANOVA, comparing to no NR within each group. H,I) Immunofluorescent images and hair cell counts showed NR restored efficiency of hair cell differentiation in PGC1α knockout cochlear explants. (Scale bar: 50 µm) Error bars represent mean ± SEM. n = 4 cochlear explants at each condition. ^**^ *P* < 0.01 by unpaired *t*‐test.

A major pathway of NAD^+^ biosynthesis in mammals is the salvage pathway,^[^ ^47^ ^]^ which is driven by the mitochondrial biogenesis regulator PGC1α.^[^ ^48^ ^]^ Deficiency of PGC1α reduces the enzymes that synthesize NAD *de novo* from amino acids.^[^ ^48^ ^]^ We next determined if loss of PGC1α in supporting cells may impair hair cell differentiation via NAD^+^. To conditionally knockout Pgc1α in cochlear supporting cells, we crossed floxed Pgc1α (Pgc1α^fl/fl^) mice with two tamoxifen‐inducible Cre mice, Sox2‐CreER (all cochlear supporting cells) and Lgr5‐EGFP‐IRES‐CreER (cochlear progenitor cells). Conditional knockout (cKO) of PGC1α in Sox2+ supporting cells was validated by RT‐qPCR in cultured cochlear explants (**Figure** **5**A). While DAPT promoted hair cell differentiation in control Pgc1α^fl/fl^ explants, its effect was completely abolished by PGC1α‐cKO (Figure 5B). PGC1α‐cKO explants also showed increased mitochondrial ROS levels, which may be further induced by DAPT treatment and scavenged by the antioxidant NAC (Figure 5C). As expected, the ROS scavenger NAC restored hair cell differentiation by DAPT treatment in the PGC1α‐cKO explants (Figure 5D). Importantly, PGC1α‐cKO significantly reduced the NAD^+^/NADH ratio in cochlear organoids (Figure 5E), consistent with its pivotal role in NAD^+^ biogenesis.^[^ ^48^ ^]^ Impaired hair cell differentiation was also observed in cochlear organoids derived from PGC1α‐cKO mice (Figure 5F). To determine if NAD^+^ levels contributed to hair cell differentiation in PGC1α‐cKO tissues, we then co‐treated both organoids and explants with the NAD^+^ precursor NR. Indeed, the ratio of Myo7a+ organoids derived from PGC1α‐cKO cochlea was increased by NR in a dose‐dependent manner (Figure 5G). Similarly, hair cell differentiation in the PGC1α‐cKO cochlear explants was restored by NR co‐treatment (Figure 5H,I). Similar results were obtained from cochlear organoids with PGC1α‐cKO from Lgr5+ supporting cells (Figure S7A, Supporting Information). While PGC1α‐cKO did not affect the proliferation of Lgr5+ organoids (Figure S7B, Supporting Information), it significantly reduced the ratio of differentiated Myo7a+ organoids (Figure S7C,D, Supporting Information). Together, the data support the notion that NAD^+^ plays an essential role in cochlear hair cell differentiation.

### 2.6 α‐KG and NAD^+^ Promote Atoh1‐Induced Hair Cell Reprogramming In Vivo

Atoh1, a bHLH transcription factor, is critical for the development and regeneration of hair cells.^[^ ^49^, ^50^ ^]^ However, induced differentiation of cochlear hair cells by Atoh1 overexpression appears to be limited to the greater epithelial ridge (GER) supporting cells, while the pillar and Deiters’ cells at the lesser epithelial region (LER) are rarely reprogramed to hair cells despite their potent expression of the exogenous Atoh1.^[^ ^51^, ^52^, ^53^, ^54^, ^55^ ^]^ Therefore, we first tested if α‐KG co‐treatment could promote hair cell differentiation at LER in cochlear explants with Atoh1 overexpression in Sox2+ supporting cells (Atoh1‐cOE). As expected, the induced hair cells (iHCs) at LER were scarce in the Atoh1‐cOE explants (Figure S8A,B, Supporting Information). However, α‐KG co‐treatment significantly increased Atoh1‐HA/Pou4f3 double positive iHCs around the OHC region (Figure S8, Supporting Information).

**Figure 6:**
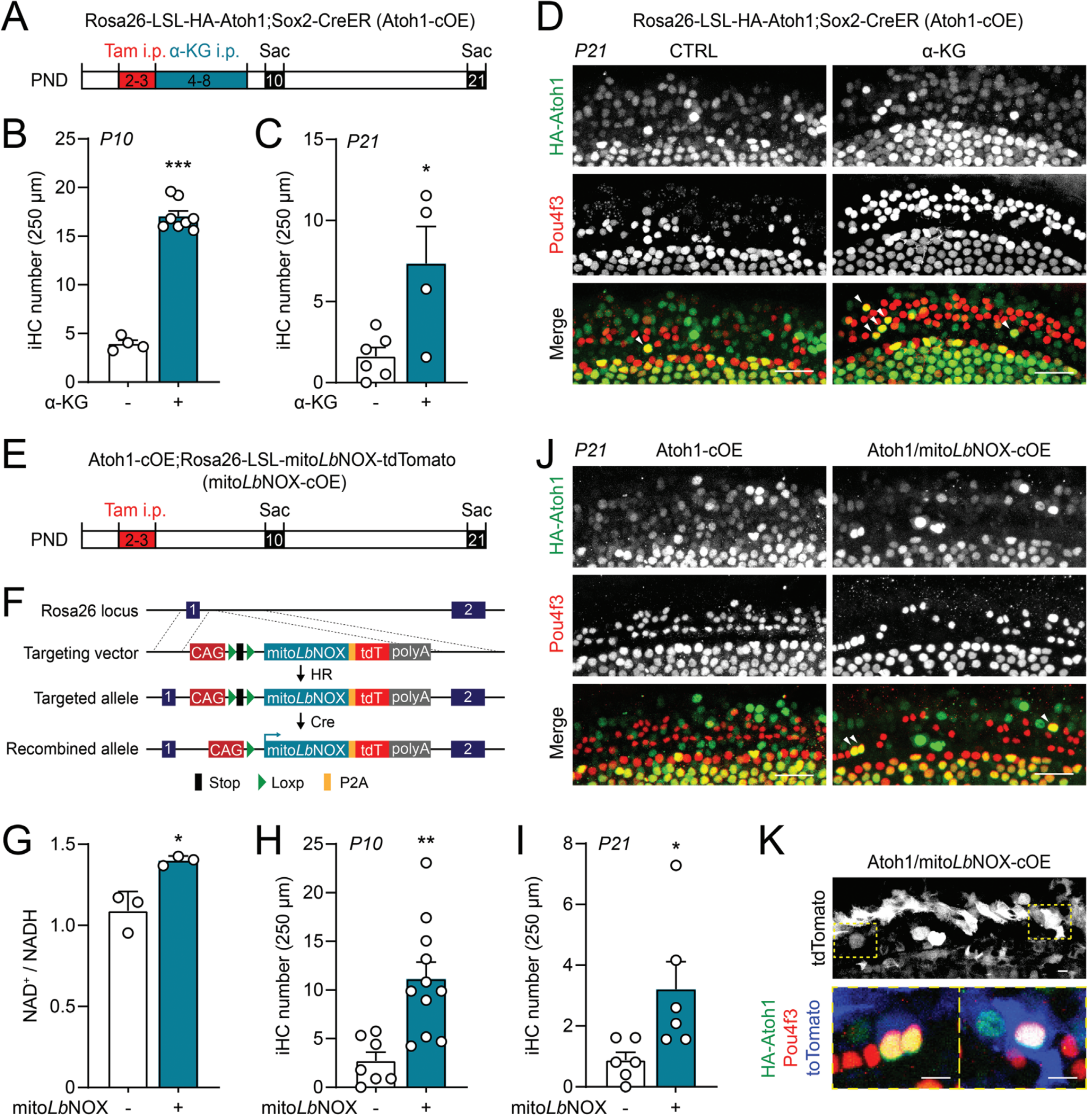
α‐KG and NAD^+^ promote Atoh1‐induced hair cell differentiation in vivo. A) Flow chart of α‐KG in vivo experiment. PND, postnatal day; I.P. intraperitoneal; Tam, tamoxifen; Sac, sacrifice. B,C) α‐KG treatment increased Atoh1‐induced hair cells (iHC) at P10 and P21. iHC was defined by HA‐Atoh1/Pou4f3 double positive hair cells. Error bars represent mean ± SEM. n = 4–8 mice at each condition. ^*^ *P* < 0.05 and ^***^ *P* < 0.001 by unpaired *t*‐test. D) Confocal images of cochlear wholemounts from P21 Atoh1‐cOE mice with or without α‐KG treatment. Arrowheads showed induced hair cells at OHC/LER area. (Scale bars: 20 µm) E) Flow chart of mito*Lb*NOX in vivo experiment. F) Targeting strategy for generating Rosa26‐LSL‐mito*Lb*NOX‐tdTomato mice. HR, homologous recombination. G) Increased NAD^+^/NADH ratio in mito*Lb*NOX;Sox2‐CreER cochlear organoids. Sox2‐CreER organoids were used as control. Error bars represent mean ± SD. n = 3 biological replicates with each replicate containing 200–300 organoids. ^*^ *P* < 0.05 by unpaired *t*‐test. H,I) mito*Lb*NOX co‐expression increased Atoh1‐induced hair cells (iHC) at P10 and P21. iHC was defined by HA‐Atoh1/Pou4f3 double positive hair cells. Error bars represent mean ± SEM. n = 6–11 mice at each condition. ^*^ *P* < 0.05 and ^**^ *P* < 0.01 by unpaired *t*‐test. J) Confocal images of cochlear wholemounts from P21 Atoh1‐cOE or Atoh1/mito*Lb*NOX‐cOE mice. Arrowheads showed induced hair cells at the OHC/LER area. (Scale bars: 20 µm) K) Co‐labeling of iHCs with mito*Lb*NOX (tdTomato). (Scale bars: 5 µm).

We next examined the effects of α‐KG on Atoh1‐cOE mice in vivo by I.P. administration of α‐KG daily from P4‐8 (**Figure** **6**A). Remarkably, iHCs at the LER region were also significantly increased by α‐KG administration at both P10 (Figure 6B) and P21 (Figure 6C,D), consistent with the data from cochlear explants (Figure S8, Supporting Information).

Lastly, synergistic effects of NAD^+^ and Atoh1 expression on hair cell differentiation were also examined in vivo (Figure 6E). We generated a knockin mouse model for conditional overexpression (cOE) of mito*Lb*NOX (Figure 6F). This model allows us to investigate the specific role of NAD^+^ in hair cell differentiation. Mito*Lb*NOX‐cOE in Sox2+ supporting cells significantly increased the ratio of NAD^+^/NADH (Figure 6G), similar to that observed by mito*Lb*NOX lentiviral infections (Figure 4D). Consistently, co‐overexpression of Atoh1 and mito*Lb*NOX promoted hair cell reprogramming at the LER region at both P10 (Figure 6H) and P21 (Figure 6I,J). Triple labeling of HA‐Atoh1, mito*Lb*NOX (tdTomato) and Pou4f3 was evident in the P21 cochlea overexpressing both Atoh1 and mito*Lb*NOX (Figure 6K). Together, our findings reveal the important roles of the key metabolites, α‐KG and NAD^+^, in hair cell differentiation in vitro and in vivo.

## 3 Discussion

Direct reprogramming of the cochlear supporting cells into functional hair cells represents one of the most promising approaches to treating hearing loss.^[^ ^7^, ^8^, ^9^ ^]^ However, cellular processes affecting the reprogramming efficiency are not completely understood. Based on the metabolomic profiles of the expanding and differentiated cochlear organoids, we propose that the decoupling between tricyclic acid (TCA) cycle and electron transport chain (ETC) not only accompanies but also impairs the reprogramming of hair cells from supporting cells (Figure S9, Supporting Information). Our study also highlights the importance of key metabolic factors, such as PGC1α, α‐KG, and NAD^+^ to efficient hair cell reprogramming.

While metabolic rewiring is known to play critical roles in the differentiation and fate determination of neurons, myocytes, and hepatocytes,^[^ ^13^, ^16^, ^17^ ^]^ whether and how it is involved in hair cell differentiation is previously unknown. Recently, studies in nonmammalian vertebrates have started to unveil the involvement of energy metabolism in the development of otic vesicles and cochlear tonotopy.^[^ ^56^, ^57^ ^]^ Lactate, a metabolic product of anaerobic glycolysis, co‐signals with the EGF signaling pathway to regulate the expression of otic lineage genes in zebrafish.^[^ ^56^ ^]^ In addition, glucose metabolism may also regulate the developmental morphogen gradient that is required for tonotopic identity of chicken basilar papilla hair cells.^[^ ^57^ ^]^ Our study suggests that differences in energy metabolism also exist in mammalian cochlear hair cells and supporting cells. Hair cell differentiation is associated with increased mitochondrial respiration (Figure 1) and upregulation of TCA enzymes (Figure S6, Supporting Information). Together with the enriched expression of TCA enzymes in adult cochlear hair cells, it is likely that the metabolic demand is higher in hair cells than in the surrounding supporting cells. Therefore, for efficient hair cell reprogramming from the supporting cells, rewiring of the metabolic status should be warranted.

Our study also provides novel knowledge of the metabolic alterations during mammalian hair cell differentiation. Unlike the cochlear hair cells, supporting cells are low in mitochondrial ETC activity (oxygen consumption). During reprogramming, the differentiating supporting cells upregulate mitochondrial respiration, resulting in redox unbalance and ROS production, which impairs hair cell differentiation. Under homeostatic conditions, cellular oxidoreduction is typically well balanced by the NAD(P)^+^ and NAD(P)H.^[^ ^33^ ^]^ The unbalanced oxidoreduction during hair cell reprogramming is likely caused by the decoupling between increased ETC activity (ROS‐producing) and reduced TCA intermediates (NAD^+^ producing). This notion is supported by increased ROS production and decreased hair cell reprogramming in PGC1α‐deficient supporting cells, which can be remediated by treatments with either ROS scavenger or NAD^+^ precursor.

Among the reduced TCA intermediates, α‐KG is known to be involved in the differentiation processes of germ and tissue stem cells.^[^ ^40^, ^41^, ^42^, ^43^ ^]^ Our study confirms the similar important roles of α‐KG in hair cell differentiation regardless of the inducing signals (DAPT, VEGFRi, or Atoh1‐cOE). Although α‐KG is a multi‐faceted molecule involved in anti‐oxidative defense, energy production, cellular signaling, and epigenetic modifications,^[^ ^39^ ^]^ its effect in hair cell reprogramming is at least partially mediated by NAD^+^. NAD^+^, initially identified as a metabolic cofactor, also acts as a pivotal co‐substrate for proteins regulating metabolism and longevity.^[^ ^58^ ^]^ Similarly, NAD^+^ is intricately involved in the fates of stem cells and differentiation of neurons.^[^ ^59^, ^60^ ^]^ In our study, treatment of α‐KG induces the production of NAD^+^, which promotes hair cell differentiation both in vitro and in vivo. However, as both α‐KG and NAD^+^ also serve as epigenetic cofactors for DNA methylation and histone acetylation,^[^ ^58^, ^61^ ^]^ whether they play both metabolic and epigenetic roles in hair cell differentiation remains to be explored.

## 4 Experimental Section

### Reagents and Primers

All details of regents, chemicals, antibodies, and primers are listed in Table S1 (Supporting Information).

### Mouse Strains

*Lgr5‐EGFP‐IRES‐CreER* (#008875), *Sox2‐CreER* (#017593), and *PGC1α^fl/fl^* (#009666) mice were obtained from the Jackson Laboratory. *Rosa‐LSL‐HA‐Atoh1* mice were obtained from Dr Jian Zuo laboratory. C57BL6 wildtype and *CAG‐LSL‐mitoLbNOX‐P2A‐tdTomato‐PolyA* knockin mice were obtained from Gempharmatech, China and the National Resource Center for Mutant mice of China, respectively. Both male and female mice on C57BL6 backgrounds were used in this study. All animal procedures were approved by the Institutional Animal Care and Use Committee of the Model Animal Research Center of Nanjing University, China with protocol approval number #WGQ05.

### 3D Cochlear Organoid Culture

Cochlear sensory epithelia were isolated from postnatal day 3 (P3) mice and were then treated with thermolysin to separate the cochlear epithelium from the underlying mesenchyme. Sensory epithelia were then treated with TrypLE containing 200 units mL^−1^ DNases I to obtain single cells. Single cells were filtered through a 40 µm filter and then re‐suspended into a 96‐well ultra‐low attachment surface plate in culture medium with 2% Matrigel and 0.02 mg mL^−1^ laminin. The culture medium consists of serum‐free 1:1 mixture of DMEM and F12, supplemented with Glutamax I, N2, B27, murine EGF (50 ng mL^−1^), murine FGF (50 ng mL^−1^), murine IGF‐1 (50 ng mL^−1^) and small molecules, including CHIR99021, VPA, pVc and 616452. Cultured organoids were split 1:2 every two days. At day 10 in vitro (DIV10), these organoids were switched to a differentiation medium consisting of a serum‐free 1:1 mixture of DMEM and F12, supplemented with Glutamax I, N2, B27, and small molecules, including CHIR99021 (3 µm) and LY411575 (10 µm). Half of the differentiation medium was replaced afresh every two days. *PGC1α^fl/fl^:Sox2‐CreER* and *PGC1α^fl/fl^:Lgr5‐EGFP‐IRES‐CreER* cochlear organoids were cultured in serum‐free 1:1 mixture of DMEM and F12, supplemented with Glutamax I, N2, B27, murine EGF (50 ng mL^−1^), murine FGF (50 ng mL^−1^), murine IGF‐1(50 ng mL^−1^) and small molecules, including CHIR99021, VPA, pVc, and 616452, in the presence of 4‐Hydroxytamoxifen (10 µm).

### Cochlear Explant Culture

Cochlear sensory epithelia were isolated from P3 mice and cultured as explants in a collagen‐coated 35 mm dish. Cochlear explants were cultured in a serum‐free 1:1 mixture of DMEM and F12, supplemented with Glutamax I, N2, B27, and Penicillin G. After 24 h, fresh culture medium was replaced without growth factors, and with CHIR99021 (3 µm) and small molecules (DAPT, NR, NAC, Creatine or α‐KG). Fresh medium was replaced every two days until sample collection and analysis.

### Hypoxia Treatment

Cochlear organoids at the differentiating stage (DIV10‐20) and cochlear explants were either cultured in a normal oxygen environment (normoxia) or in a cell culture incubator supplemented with nitrogen to maintain 5% oxygen concentration (hypoxia). Change of media was carried out at a minimal time period in a normal atmosphere. Organoids or explants were maintained in normoxia or hypoxia throughout the differentiation stage.

### Immunofluorescence

Cochlear organoids or explants were washed three times with PBS, and then fixed at room temperature with 4% paraformaldehyde/PBS for 20 min and then washed three times with PBS. Permeabilization and blocking were carried out in a blocking buffer (0.3% Triton X‐100 and 5% heat‐inactivated normal horse serum in PBS) for 1 h at room temperature. Organoids or explants were then incubated with diluted primary antibodies (0.3% Triton X‐100 and 1% heat‐inactivated normal horse serum in PBS) overnight at 4 °C. Hair cells were immuno‐stained by markers including Pou4f3 and Myo7a, HA‐Atoh1 mice were immuno‐stained by HA‐tag marker. Secondary antibodies (Alexa Fluor 488, Alexa Fluor 568, and Alexa Fluor 647 conjugated) were used at 1:500. Nuclei were visualized by DAPI. To measure mitochondrial ROS, cochlear explant samples were washed with HBSS after 6 days of incubation and stained with 5 µm MitoSox Red diluted in PBS for 10 min in a humidified atmosphere of 5% CO_2_ at 37 °C, prior to antibody incubations.

For immunofluorescence of postnatal mouse cochlea, temporal bones were isolated and perfusion‐fixed with 4% paraformaldehyde for 2 h at room temperature, decalcified in 5% EDTA, and micro‐dissected as wholemounts. The cochlear wholemounts were then blocked and immuno‐labeled with Pou4f3 and HA‐tag antibodies. Pou4f3 was used to labeled hair cells and nuclei visualized by DAPI. For immunofluorescence of frozen sections from wild‐type mice, the samples were immuno‐labeled with Mpc1, Mpc2, Aco2, Dlst, and Sdha antibodies, and nuclei visualized by DAPI. All imaging was carried out with Leica SP5 confocal microscope (Leica, Germany).

### Western Blot Analyses

Cochlear organoid tissues were cultured and treated as described. Total protein from 30 well cochlear organoids was extracted in 20 µL lysis buffer (50 mm Tris‐HCl, pH 7.4, 150 mm NaCl, 1 mm EDTA, pH 8.0, 1% Triton X‐100) with 1 mm PMSF and proteinase inhibitor cocktail (Roche, Germany). The lysates underwent freeze‐thaw followed by incubation at 95 °C for 20 min in a protein loading buffer. Samples were separated by 10% SDS‐PAGE and followed by western blot analysis using antibodies against Sdha and beta‐actin (Actin). Blot signals were detected and analyzed using the Tanon infrared imaging system (Tanon, China). Densitometry was performed using ImageJ software (version 1.46r, NIH, MD).

### RNA Extraction and RT‐qPCR

Organoids were collected in a 1.5 mL centrifuge tube and centrifuged at 1000 rpm for 4 min. The pelleted organoids were then added to 1 mL RNAiso Plus (Takara, Japan). Total RNA was isolated as per the manufacturer's instructions. Reverse transcription was performed using PrimeScript RT reagent Kit (Vazyme,China). SYBR Green qPCR was performed in duplicates using Hieff qPCR SYBR Green Master Mix (No Rox) kit (Yeasen, China). Gapdh was used as a reference gene. Details of the primers are listed in Table S1 (Supporting Information).

### RNA‐seq Experiments and Analyses

Total RNA from the cochlear organoids was extracted using RNAiso Plus. Total RNA quality was determined by 2100 Bioanalyser (Agilent) and quantified using the ND‐2000 (NanoDrop Technologies). Only high‐quality RNA sample (OD260/280 = 1.8≈2.2, OD260/230 ≥ 2.0, RIN ≥ 6.5, 28S:18S ≥ 1.0, >2 µg) was used to construct sequencing library.

RNA purification, reverse transcription, library construction, and sequencing were performed at Majorbio Bio‐pharm Biotechnology Co., Ltd. (Shanghai, China) according to the manufacturer's instructions (Illumina, San Diego, CA). RNA‐seq transcriptome library was prepared following TruSeq RNA sample preparation Kit from Illumina (San Diego, CA) using 1 µg of total RNA. Shortly, messenger RNA was isolated according to the polyA selection method by oligo(dT) beads and then fragmented by fragmentation buffer first. Second double‐stranded cDNA was synthesized using a SuperScript double‐stranded cDNA synthesis kit (Invitrogen, CA) with random hexamer primers (Illumina). Then the synthesized cDNA was subjected to end‐repair, phosphorylation, and “A” base addition according to Illumina's library construction protocol. Libraries were size selected for cDNA target fragments of 200–300 bp on 2% Low Range Ultra Agarose followed by PCR amplified using Phusion DNA polymerase (NEB) for 15 PCR cycles. After quantified by TBS380, the paired‐end RNA‐seq sequencing library was sequenced with the Illumina Novaseq 6000 (2 × 150 bp read length). The raw paired‐end reads were trimmed and quality controlled by SeqPrep and Sickle with default parameters. Then clean reads were separately aligned to the reference genome with orientation mode using TopHat (version 2.1.1) software.^[^ ^62^ ^]^ The mapping criteria of bowtie was as follows: sequencing reads should be uniquely matched to the genome allowing up to two mismatches, without insertions or deletions. Then the region of the gene was expanded following depths of sites and the operon was obtained. In addition, the whole genome was split into multiple 15 kb windows that share 5 kb. New transcribed regions were defined as more than two consecutive windows without the overlapped region of the gene, where at least two reads were mapped per window in the same orientation.

To identify DEGs (differential expression genes) between two different samples, the expression level of each transcript was calculated according to the fragments per kilobase of exon per million mapped reads (FPKM) method. RSEM was used to quantify gene abundances.^[^ ^63^ ^]^ R statistical package software EdgeR was utilized for differential expression analysis.^[^ ^64^ ^]^ In addition, functional‐enrichment analysis including GO and KEGG was performed to identify which DEGs were significantly enriched in GO terms and metabolic pathways at Bonferroni‐corrected P‐value ≤0.05 compared with the whole‐transcriptome background. The Gene Ontology (GO) analysis will be done with DAVID GO Annotation. Important gene lists of different signaling pathways will be determined based on information from the KEGG database. The RNA‐seq data were available in GEO database with accession number GSE243581.

### Oxygen Consumption Rate Measurements

Oxygen consumption rates were measured in cochlear organoids (expansion and differentiation stages) using a 24‐well Seahorse XF24 Islet Capture Microplate Analyzer. Cochlear organoids were cultured for 10 days (expansion) and 20 days (differentiation) in 96‐well plates. Organoids were collected in a centrifuge tube, and washed three times in Base Medium (Seahorse XF DMEM Medium, supplemented with 20 mm glucose, 5 mm sodium pyruvate, and 2 mm glutamine, pH 7.4). Organoids were plated in XF24 microplates (30–60 organoids per well) and incubated in a CO_2_‐free incubator with Base Medium for 1 h. Oxygen consumption rate (OCR) was measured at baseline (Oligomycin, 5 µm) and after the addition of respiratory inhibitor FCCP (5 µm, which uncouples oxidative phosphorylation from membrane potential), followed by rotenone/antimycin A (2 µm, a Complex I inhibitor). After the assay was completed, organoids were collected for protein quantification, and the OCR reading was normalized to the protein concentration of each well.

### NAD^+^/NADH Measurements

The luciferin Detection Reagent and the NAD/NADH‐Glo Detection Reagent were prepared (Promega). Cochlear organoids were collected in a centrifuge tube as each sample (200–300 organoids), washed once in PBS, and resuspended to the final volume per well at 50 µL. Cochlear organoids were lysed with 50 µL base solution (0.2N NaOH and 1% DTAB).To measure NAD^+^, 25 µL of 0.4 N HCl was added to 50 µL lysate and heated at 60 °C for 15 min. After incubating at room temperature for 10 min, 25 µL of Trizma base was added to each well of the acid‐treated sample. To measure NADH, 50 µL of lysate was heated at 60 °C for 15 min and incubated at room temperature for 10 min. HCl/Trizma solution (50 µL) was added to each well of the base‐treated sample. The cochlear organoid lysates were collected in a white‐walled plate and equilibrated at room temperature for 5 min. 50 µL of NAD/NADH‐Glo Detection Reagent was added to each well. The samples were then incubated for 60 min at room temperature, followed by luminescence recording using a luminometer.

### Non‐Targeted Metabolomics

Metabolomic analysis was performed in collaboration with LipidALL Technologies. Reagents were prepared as follows: water was purified using an ultrapure water preparation system; LC‐MS grade acetonitrile and methanol were purchased from Merck (Germany); HPLC grade formic acid was obtained from Sigma (Germany). For metabolome extraction, 40 wells of organoids at each condition were pulled as one sample. Samples were washed with ice‐cold PBS three times for 1 min at 1000 g and 4 °C. Samples were mixed with ice‐cold 80% methanol in water, and incubated for 30 min at 1500 rpm and 4 °C. Samples were then centrifuged for 10 min at 12 000 rpm and 4 °C. The supernatants were removed into clean 1.5 mL centrifuge tubes and dried using SpeedVac (Genevac miVac, Tegent Scientific Ltd., England).

The dried extracts were re‐dissolved with 1% acetonitrile in water, and upper‐layer liquids were collected for LC‐MS analysis. The ACQUITY UPLC HSS T3 1.8 µm, 3.0 × 100 mm columns (Waters, Dublin, Ireland) were used for reversed‐phase chromatographic analysis. Ultra‐performance Liquid Chromatography (Agilent 1290 II, Agilent Technologies, Germany) coupled to Quadrupole‐TOF MS (5600 Triple TOF Plus, SCIEX, Singapore) was applied to acquire metabolome data. The MS parameters for detection were ESI source voltage was set at 5.5 kV in positive ion mode, and −4.5 kV in negative ion mode; vaporizer temperature, 500 °C; drying gas (N2) pressure, 50 psi; nebulizer gas (N2) pressure, 50 psi; curtain gas (N2) pressure, 35 psi; The reversed‐phase chromatographic analysis scan range was *m/z* 60–700. Information‐dependent acquisition mode was used for MS/MS analyses of the metabolites. The collision energy was set at (‐) 35–15 eV. Data acquisition and processing were performed using Analyst TF 1.7.1 Software (AB Sciex, Concord, ON, Canada).

For data processing, all detected ions were extracted using MarkerView 1.3 (AB Sciex, Concord, ON, Canada) into Excel in the format of 2D matrix, including mass‐to‐charge ratio (m/z), retention time, and peak areas, and isotopic peaks. PeakView 2.2 (AB Sciex, Concord, ON, Canada) was applied to extract MS/MS data and perform a comparison with the Metabolites database (AB Sciex, Concord, ON, Canada), HMDB, METLIN, and standard references to annotate ion ID. Self‐compiled R program was used for statistical analysis. The concentration of each metabolite was normalized to the total metabolite quantity of each sample.

### Molecular Cloning and Lentiviral Infections

*MitoLbnox* was cloned into lentiviral constructs of pLKO.1 vector (Addgene 10879). Lentiviruses were produced by transfection of lentiviral backbones containing the indicated transgenes together with packaging plasmids pSPAX2 (Addgene 12260) and pMD2G (Addgene 12259) into HEK293T cells (ATCC R CRL‐3216TM). Viruses were concentrated from culture supernatant by ultra‐centrifugation (25,000 rpm, 2 h, 4 °C). After 24–48 h infection with 10 µg mL^−1^ polybrene in organoid 3D culture, the virus‐containing medium was replaced with fresh culture media.

### Statistical Analysis

Statistical tests were performed using GraphPad Prism 9. Results were reported as mean ± SEM. Replicate values in all experiments were from biological replicates of 2–5 independent experiments and the numbers are as indicated by individual data points in each graph. Each biological sample was randomly allocated to control or different treatment groups. The specific statistical tests used in each experiment were described in figure legends. The sample size was determined empirically according to previous experience in the research field.

## Conflict of Interest

The authors declare no conflict of interest.

## Author Contributions

Q.L. and L.Z. contributed equally to this work. G.W. supervised the work; Z.G., X.G., and G.W. designed the work; Q.L., L.Z., Z.C., Y.H., Y.H., C.Q., and C.Z. performed research; D.Z. and Z.G. contributed new reagents/analytic tools; Q.L. and G.W. analyzed data; Q.L. and G.W. wrote the paper with help from other authors.

## Supporting information

Supporting Information

## Data Availability

The RNA‐seq data were available at GEO database with accession number GSE243581.
