# Supplementary material for: Metabolic Profiling of Cochlear Organoids Identifies α‐Ketoglutarate and NAD+ as Limiting Factors for Hair Cell Reprogramming
Source: Adv Sci (Weinh). 2024 Jul 11;11(34):2308032. doi: 10.1002/advs.202308032 (PMC11425867; doi:10.1002/advs.202308032)
Supplement: Supplementary file 1 — Supporting Information [file ADVS-11-2308032-s001.pdf]

ADVANCED SCIENCE
Open Access

Supporting Information

for *Adv. Sci.*, DOI 10.1002/advs.202308032

Metabolic Profiling of Cochlear Organoids Identifies α-Ketoglutarate and NAD^+ as Limiting Factors for Hair Cell Reprogramming

Qing Liu, Linqing Zhang, Zhen Chen, Yihan He, Yuhang Huang, Cui Qiu, Chengwen Zhu, Danxia Zhou, Zhenji Gan, Xia Gao* and Guoqiang Wan**

SUPPLEMENTAL FIGURES AND TABLE

Metabolic profiling of cochlear organoids identifies α-ketoglutarate and NAD+ as limiting factors for hair cell reprogramming

Qing Liu[1,2,3], Linqing Zhang[1,2], Zhen Chen[1,2], Yihan He[1,2], Yuhang Huang[1,2], Cui Qiu[1,2], Chengwen Zhu[1,3], Danxia Zhou[2], Zhenji Gan[2,*], Xia Gao[1,3,*] and Guoqiang Wan[1,2,3,*]

[1]State Key Laboratory of Pharmaceutical Biotechnology, MOE Key Laboratory of Model Animal for Disease Study and Jiangsu Provincial Key Medical Discipline (Laboratory), Department of Otolaryngology Head and Neck Surgery, Affiliated Drum Tower Hospital of Medical School, Model Animal Research Center of Medical School, Nanjing University, Nanjing 210032, China.

[2]State Key Laboratory of Pharmaceutical Biotechnology, MOE Key Laboratory of Model Animal for Disease Study and Jiangsu Key Laboratory of Molecular Medicine, Model Animal Research Center of Medical School, Nanjing University, Nanjing 210032, China.

[3]Research Institute of Otolaryngology, No. 321 Zhongshan Road, 210008, Nanjing, China.

*Correspondence: ganzj@nju.edu.cn; gaoxia@nju.edu.cn or guoqiangwan@nju.edu.cn

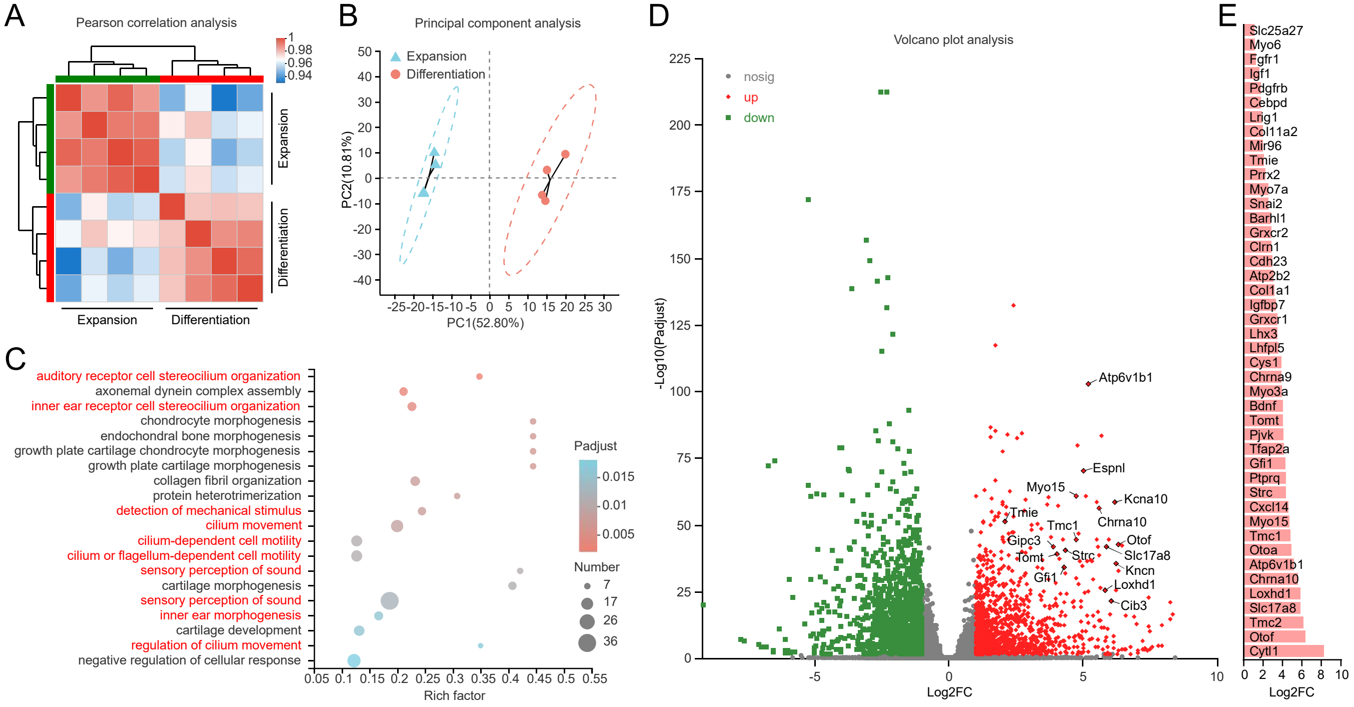

Figure S1. Upregulation of hair cell markers during cochlear organoid differentiation by transcriptomic analyses.

(A, B) Pearson correlation analysis (A) and principal component analysis (B) of RNA-seq results from cochlear organoids at expansion (DIV10) and differentiation (DIV20) stages.

(C) GO enrichment analysis of upregulated biological processes during organoid differentiation. Processes related to hair cell development and function were highlighted in red.

(D) Volcano plot analysis of up- and down-regulated genes. Known hair cell markers were annotated.

(E) Increased expression of known hair cell markers during organoid differentiation.

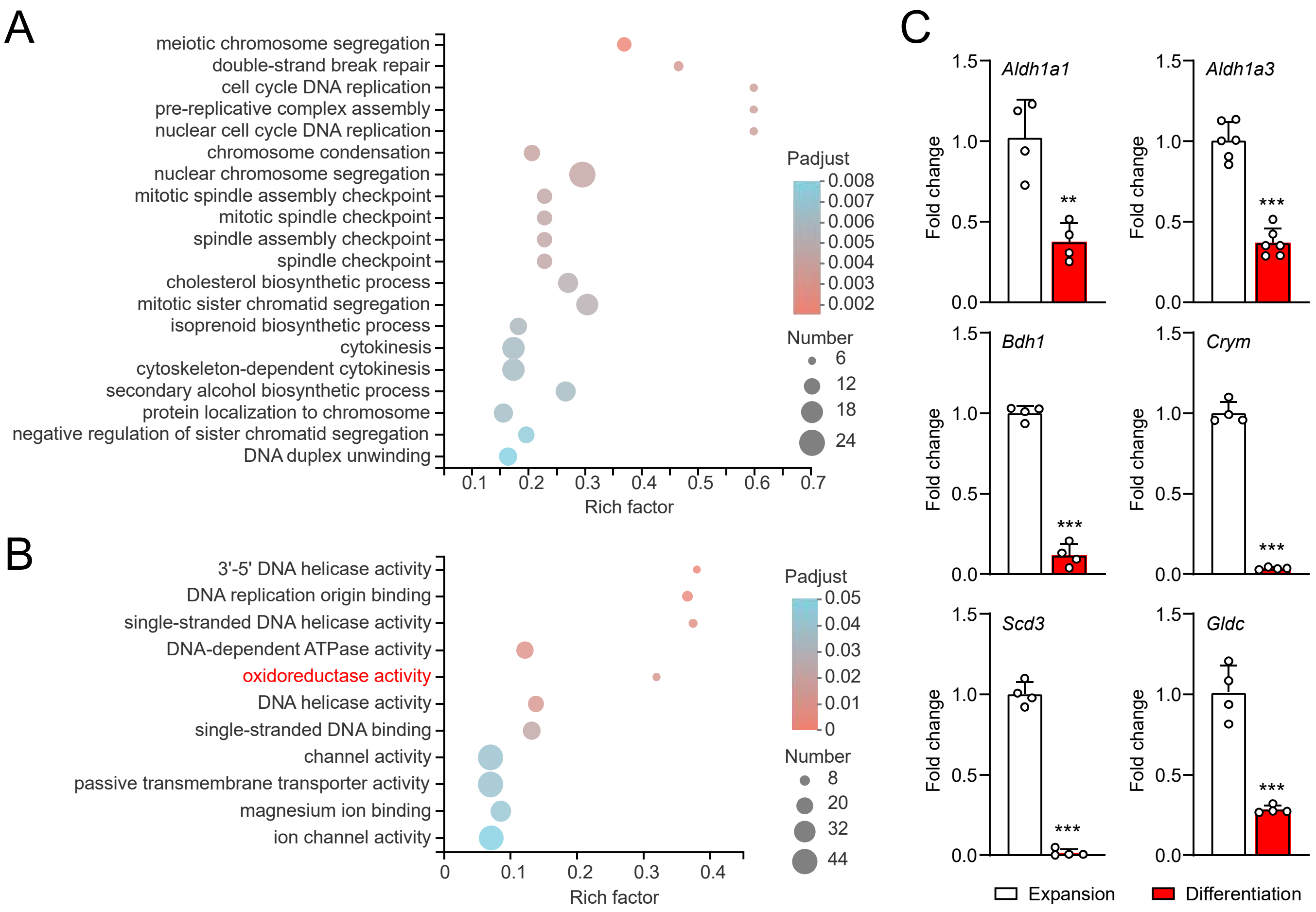

Figure S2. Downregulation of oxidoreductase activity during cochlear organoid differentiation by transcriptomic analyses.

(A, B) GO enrichment analyses of downregulated biological processes (A) and molecular functions (B) during organoid differentiation.

(C) Reduced expression of candidate genes involved in oxidoreductase activity during organoid differentiation by RT-qPCR analyses. Error bars represent mean ± SD. n = 4-6 biological replicates with each replicate containing 50-100 organoids. ** $P < 0.01$ and ** $P < 0.001$ by unpaired *t* test.

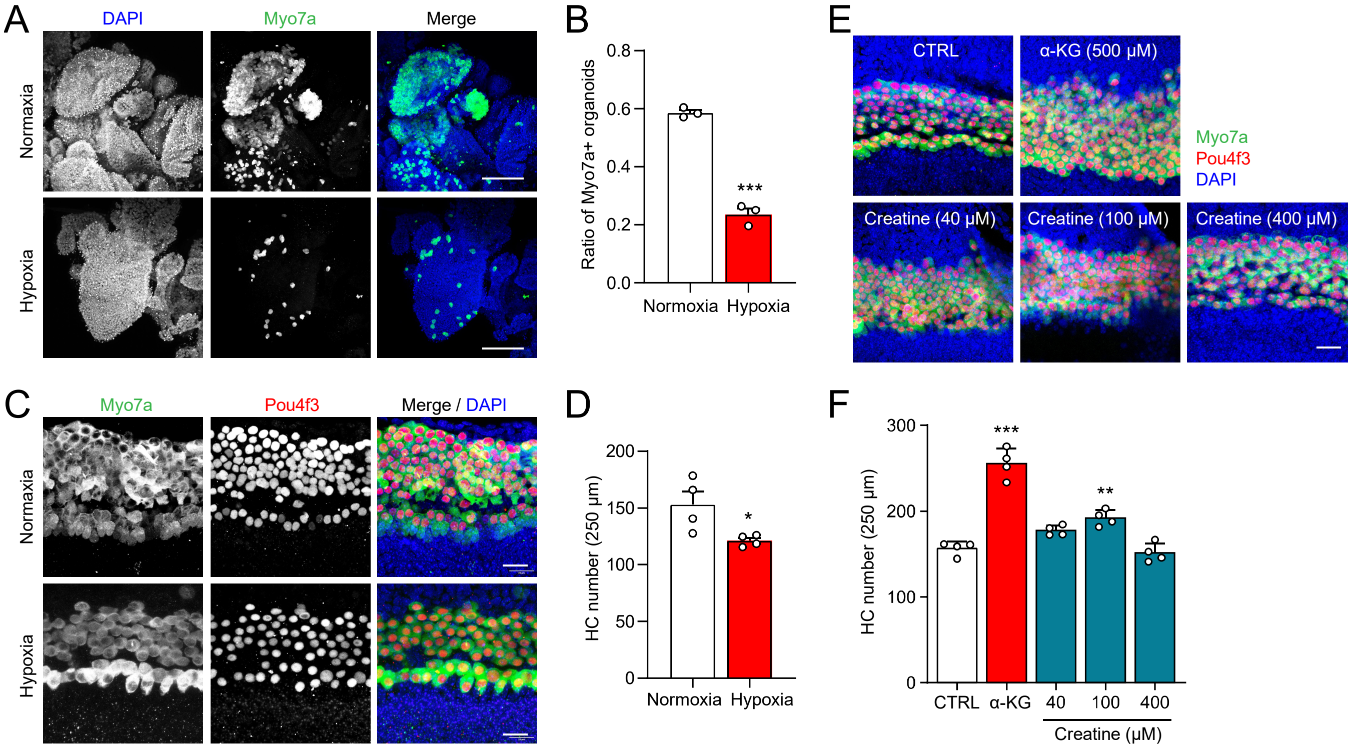

Figure S3. Effects of oxygen concentration and creatine on hair cell differentiation.

(A, B) Confocal images (A) and hair cell counts (B) show hypoxia treatment reduced efficiency of hair cell differentiation in cochlear organoids. All organoids were co-treated with 5 μM DAPT. Error bars represent mean ± SD. n = 3 biological replicates with each replicate containing 150-300 organoids. *** $P < 0.001$ by unpaired t test. (Scale bars: 100 μm)

(C, D) Confocal images (C) and hair cell counts (D) show hypoxia treatment reduced efficiency of hair cell differentiation in cochlear explants. Explants were co-treated with 5 μM DAPT. Error bars represent mean ± SEM. n = 4 cochlear explants at each condition. * $P < 0.05$ by unpaired t test. (Scale bars: 20 μm)

(E, F) Confocal images (E) and hair cell counts (F) show creatine treatment had modest effect on the efficiency of hair cell differentiation in cochlear explants. Explants were co-treated with 5 μM DAPT. Error bars represent mean ± SEM. n = 4 cochlear explants at each condition. ** $P < 0.01$ and *** $P < 0.001$ by unpaired t test. (Scale bars: 20 μm)

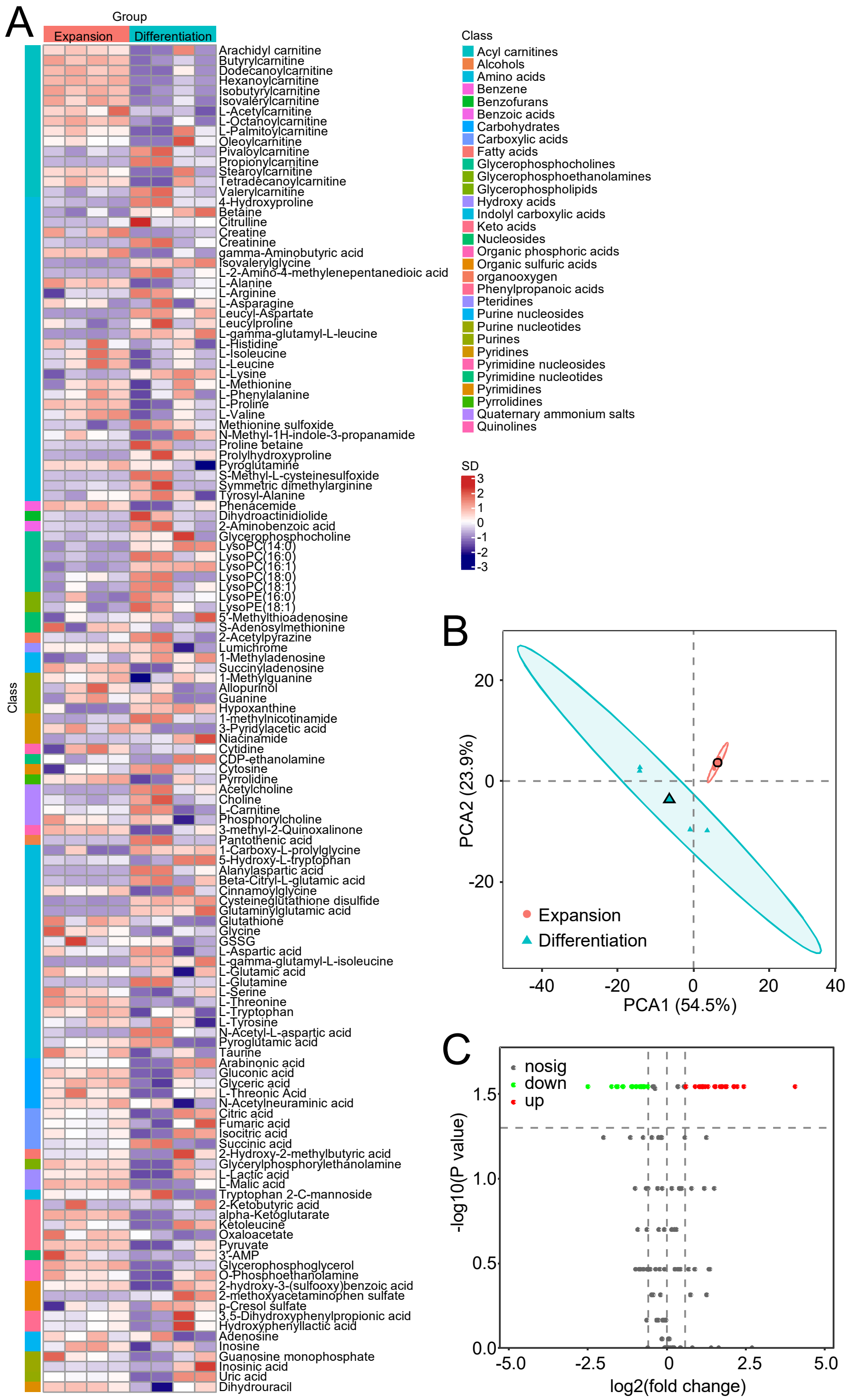

Figure S4. Non-targeted metabolomic analyses of cochlear organoids during hair cell differentiation.

(A) Heatmap analysis of differentially enriched metabolites in cochlear organoids at expansion (DIV10) and differentiation (DIV20) stages.

(B) Principal component analysis of metabolomic data from cochlear organoids at expansion and differentiation stages.

(C) Volcano plot of up- and downregulated metabolites during hair cell differentiation.

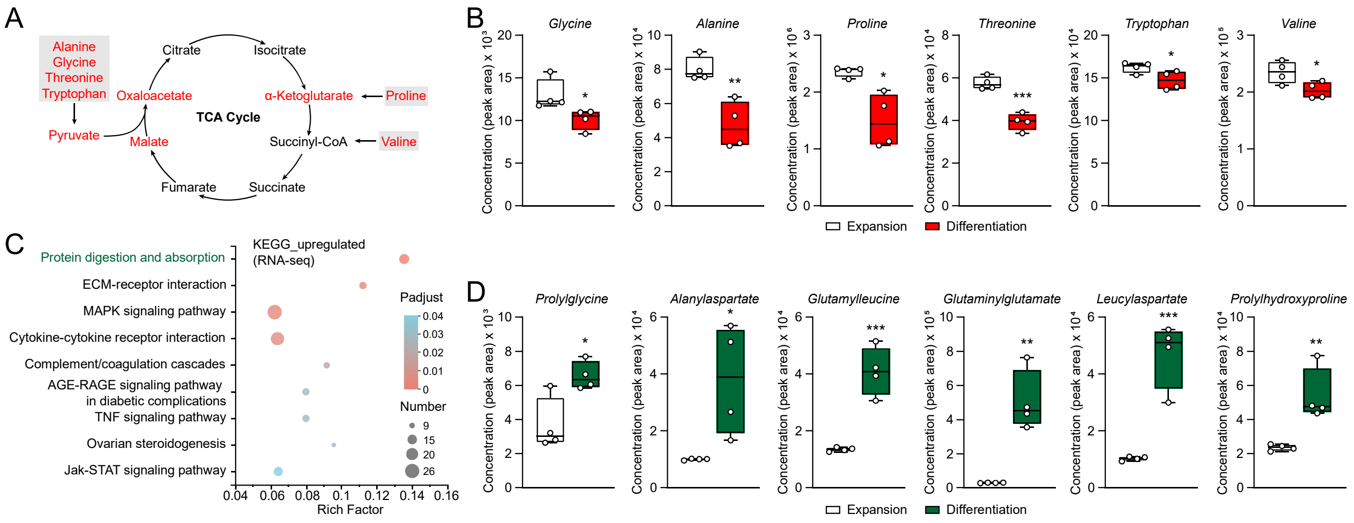

Figure S5. Decreased free amino acids and increased dipeptides during hair cell differentiation.

(A) Schematic of the TCA cycle and amino acid catabolism pathways.

(B) Concentrations of free amino acids during hair cell differentiation in cochlear organoids.

(C) KEGG enrichment analysis of upregulated cellular processes during organoid differentiation based on RNA-seq results.

(D) Concentrations of dipeptides during hair cell differentiation in cochlear organoids. (B, D) n = 4 biological replicates with each replicate containing 2000-3000 organoids. * $P < 0.05$, ** $P < 0.01$ and *** $P < 0.001$ by unpaired t test.

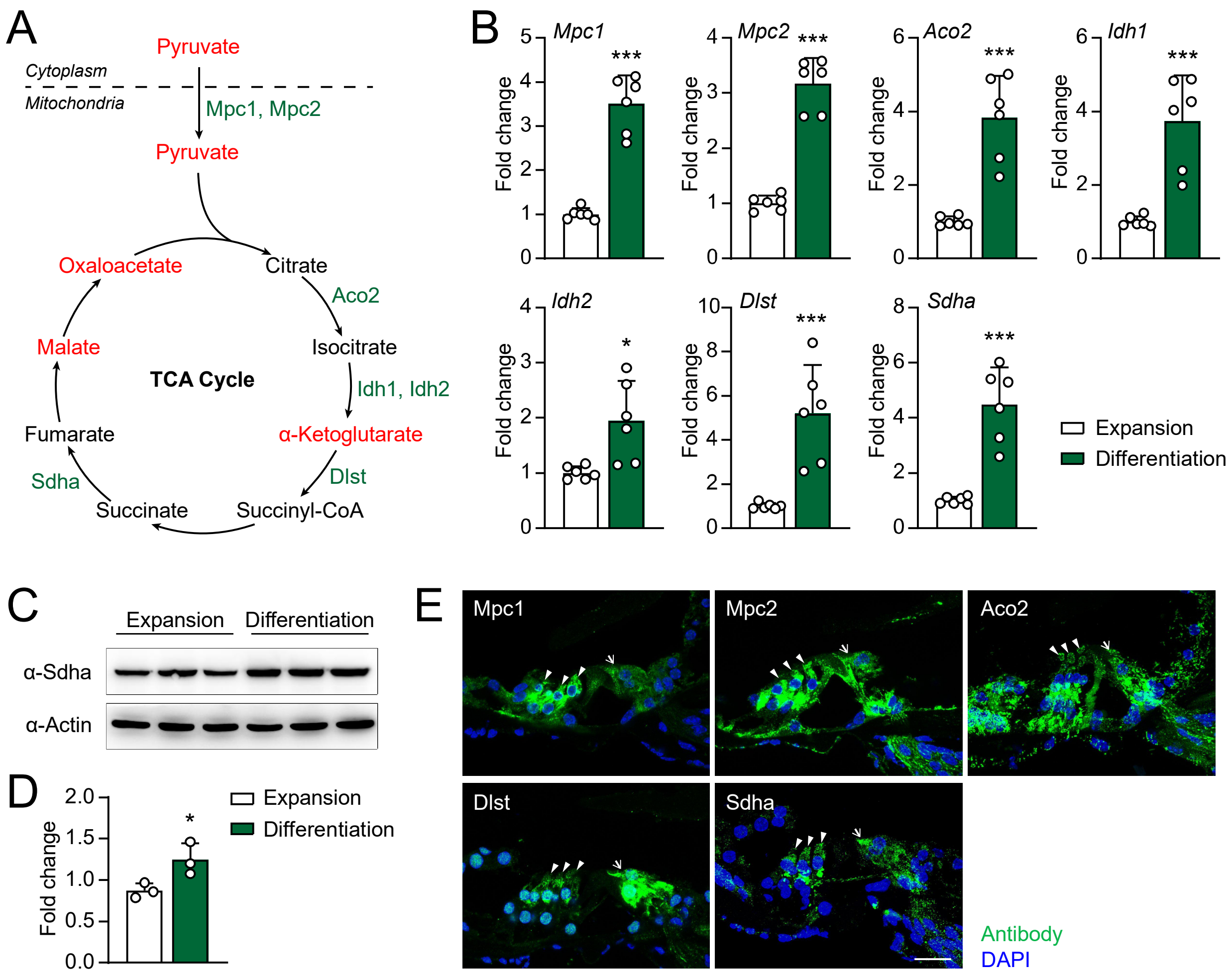

Figure S6. Increased expression of TCA cycle enzymes during hair cell differentiation and their enriched expression in mature cochlear hair cells.

(A) Schematic of TCA cycle metabolites and enzymes.

(B) Increased expression of TCA cycle enzymes during organoid differentiation by RT-qPCR analyses. Error bars represent mean ± SD. n = 6 biological replicates with each replicate containing 50-100 organoids. * $P < 0.05$ and ** $P < 0.001$ by unpaired t test.

(C, D) Western blot (C) and densitometric analysis (D) of increased Sdha protein expression during hair cell differentiation in cochlear organoids. Error bars represent mean ± SD. n = 3 biological replicates with each replicate containing 500-1000 organoids. * $P < 0.05$ by unpaired t test.

(E) Confocal immunofluorescent images of TCA cycle enzymes expressed in P21 mouse organ of Corti. Arrow and arrowheads indicate IHC and OHCs respectively. Each protein was immunolabeled by its specific antibody. (Scale bar: 20 μm)

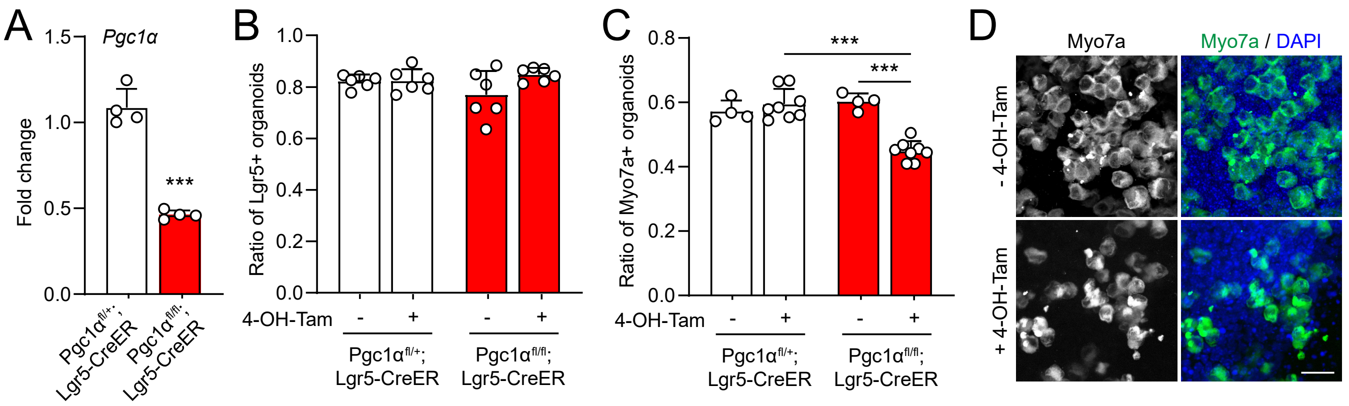

Figure S7. PGC1α knockout in Lgr5+ cells impairs hair cell differentiation.

(A) RT-qPCR validation of PGC1α (Ppargc1a) knockout in cochlear organoids derived from *PGC1α$^{fl/fl}$;Lgr5-CreER* mice. PGC1α knockout was induced by 4-OH tamoxifen treatment starting from DIV2. PGC1α$^{fl/fl}$ organoids were used as controls. Error bars represent mean ± SD. n = 4 biological replicates with each replicate containing 50-100 organoids. *** $P < 0.001$ by unpaired *t* test.

(B) PGC1α knockout does not affect expansion of Lgr5+ cochlear organoids. *PGC1α$^{fl/+}$;Lgr5-CreER* organoids were used as controls. n = 6 biological replicates with each replicate containing 150-300 organoids.

(C) PGC1α knockout in Lgr5+ cells impaired hair cell differentiation in cochlear organoids. *PGC1α$^{fl/+}$;Lgr5-CreER* organoids were used as controls. Error bars represent mean ± SD. n = 4-8 biological replicates with each replicate containing 150-300 organoids. *** $P < 0.001$ by unpaired *t* test.

(D) Confocal images of differentiated *PGC1α$^{fl/fl}$;Lgr5-CreER* cochlear organoids with (+ 4-OH-Tam) or without (- 4-OH-Tam) PGC1α knockout. (Scale bar: 20 μm)

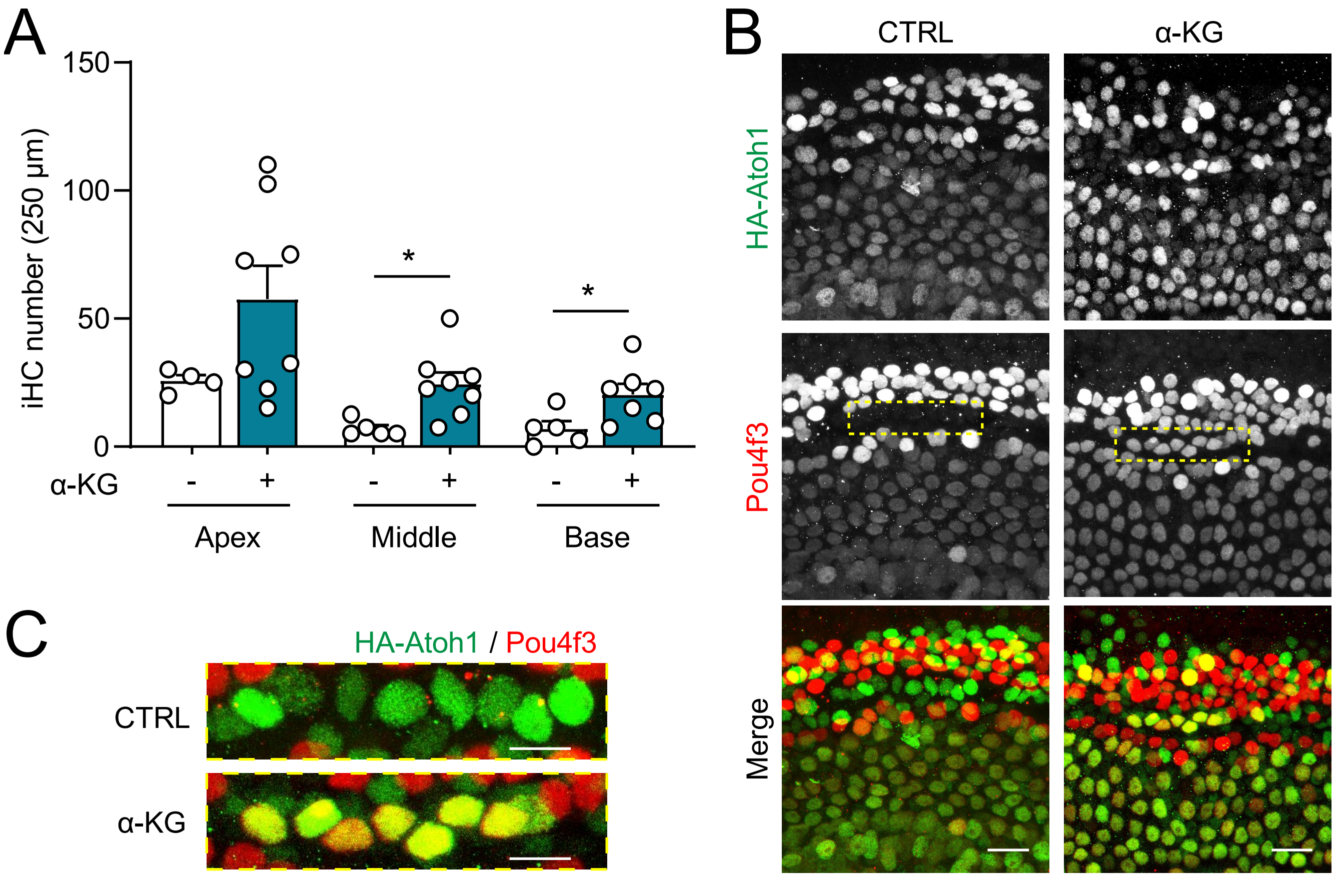

Figure S8. α-KG promotes Atoh1-induced hair cell differentiation in cochlear explants.

(A) α-KG treatment increased Atoh1-induced hair cells (iHC) at OHC/LER area. Cochlear explants from P3 *Rosa26-LSL-HA-Atoh1;Sox2-CreER* (Atoh1-cOE) mice were cultured in the absence and presence of α-KG. iHC was defined by HA-Atoh1/Pou4f3 double positive hair cells at OHC/LER area along the cochlear spiral. Error bars represent mean ± SEM. n = 4-8 cochlear explants at each condition. * $P < 0.05$ by unpaired *t* test.

(B) Confocal images of Atoh1-cOE cochlear explants treated with or without α-KG. Dotted rectangles corresponded to outer pillar cell region. (Scale bars: 20 μm)

(C) Magnified views of the dotted areas in (B) showing induced hair cells by Atoh1-expressiong and α-KG co-treatment. (Scale bars: 10 μm)

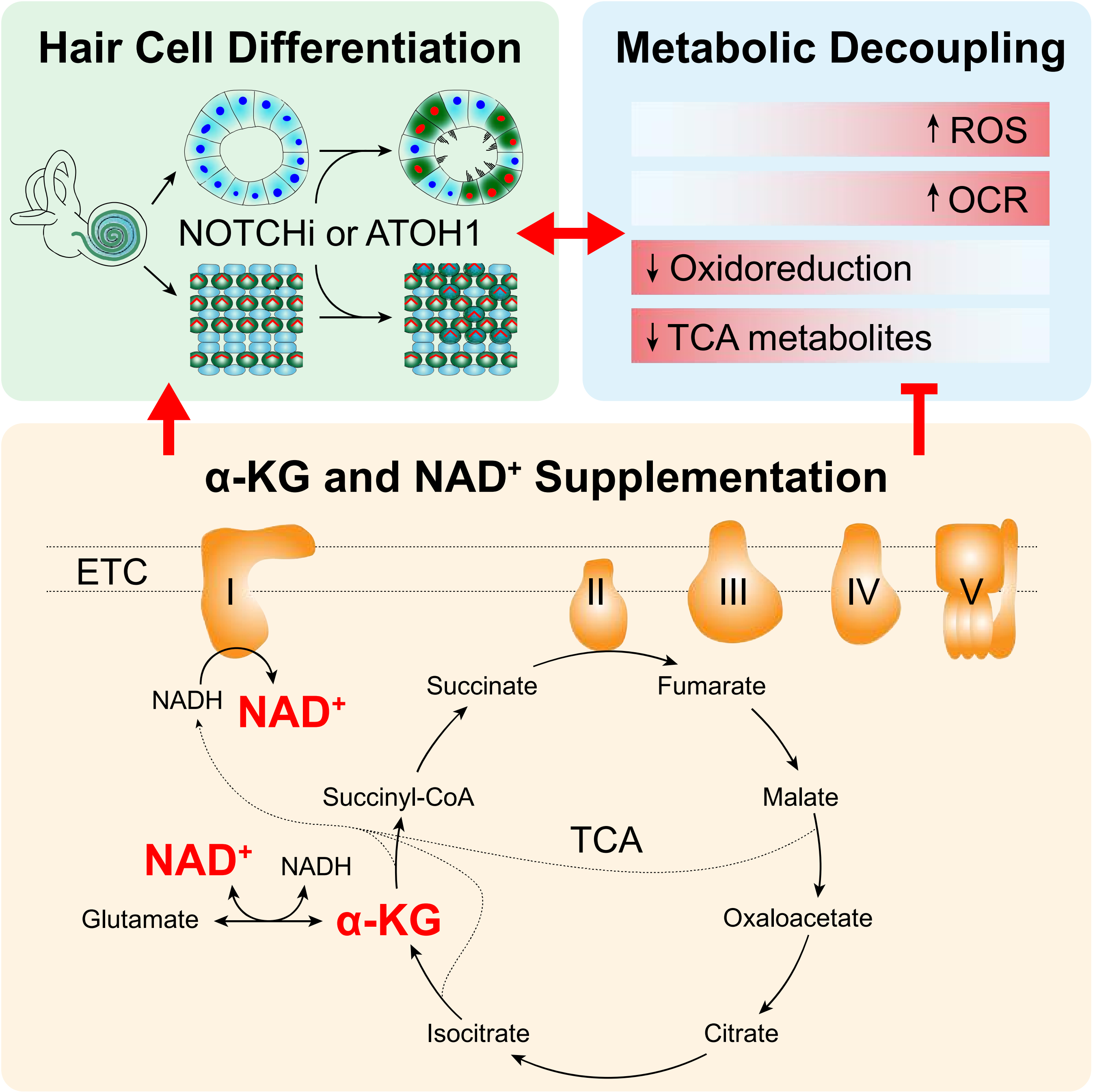

Figure S9. Graphic illustration of metabolic decoupling impairing hair cell differentiation that can be remediated by supplementation of α-KG and NAD^+.

Table S1. Details of reagents, antibodies and primers used in the study.

Key resources: regents				
**Reagent Name**	**Abbreviation**	**Final concentration**	**Vendor**	**Cat#**
Murine EGF	E	50 ng/ml (passage 1)<br>25 ng/ml (passage 2)	Peprotech	315-09
Murine FGF-basic	F	50 ng/ml	Peprotech	450-33
Murine IGF-1	I	50 ng/ml	Peprotech	250-19
CHIR99021	CHIR/C	3 µM (passage 1)<br>2 µM (passage 2)	Selleck	S1263
Creatine monohydrate	Creatine	40, 100, 400 µM	Selleck	S6023
Valproic Acid Sodium Salt	VPA/V	1 mM	Sigma	676380
2-phospho-L-ascorbic acid	pVc/P	100 µg/ml	Sigma	49752
616452	6	2 µM	Selleck	S7223
Deshydroxy LY411575	LY	10 µM	Santa Cruz	209984-56-5
Regorfenib	Reg	0.5, 1, 2, 10 µM	Selleck	S1178
DAPT	D	5, 10 µM	Santa Cruz	sc-201315

N-acetylcysteine	NAC	10, 100 µM	Selleck	S1623
Nicotinamide Riboside	NR	0.5, 1, 2, 4 mM	MCE	HY-123033
α-Ketoglutaric acid	α-KG	500 µM, 1mM	Sigma	K1128
Tamoxifen	Tmx	33 mg/kg	Sigma	T5648
Advanced DMEM/F12	-	-	Gibco	12634010
B-27 Supplement	-	-	Gibco	12587010
N2 supplement	-	-	Gibco	17502048
Glutamax I	-	-	Gibco	35050061
Penicillin G	-	2.4 mg/ml	Sigma	P3032
Laminin	-	20 ng/ml	Corning	354232
Geltrex	-	2%	Gibco	A141320
(z)-4-Hydroxytamoxifn	-	10 µM	Tocris	3412
Thermolysin	-	1 mg/ml	Sigma	P1512
TrypLE	-	-	Thermo	12604013
DNAse I	-	200 U/ml	Sigma	AMPD1
Key resources: antibodies and fluorescent molecules				
**Antibody**	**Dilution**	**Source**	**Vendor**	**Cat#**
Myosin VIIa	1:500	Rabbit Polyclonal	Proteus Biosciences	25-6790
Pou4f3	1:200	Mouse monoclonal	Santa Cruz	sc-81980

HA-Tag	1:400	Rabbit monoclonal	Cell Signaling Technology	3724
MPC1	1:200	Rabbit monoclonal	Cell Signaling Technology	14462
MPC2	1:200	Rabbit monoclonal	Cell Signaling Technology	46141
ACO2	1:200	Rabbit monoclonal	Cell Signaling Technology	6571
Dlst	1:200	Rabbit monoclonal	Cell Signaling Technology	11954
Sdha	1:200	Rabbit monoclonal	Cell Signaling Technology	11998
Beta-Actin	1:1000	Rabbit monoclonal	Cell Signaling Technology	4970
DAPI	1:2000	Fluorescent labeled nucleus	Roche	28718-90-3

Alexa Fluor 568 goat anti-mouse IgG1	1:500	Secondary antibodies	Life Technologies	A21124
488-affinipire goat anti-rabbit IgG	1:500	Secondary antibodies	Jackson	111-545-003
647-affinipire goat anti-mouse IgG	1:500	Secondary antibodies	Jackson	111-605-003
594-affinipire goat anti-mouse IgG	1:500	Secondary antibodies	Jackson	115-585-003

Key resources: qPCR primers

Gene	Forward primer	Reverse primer
Pgc1a	TGTGCTGCTCTGGTTGGT	GCTCATTGTTGTACTGGTTGGAT
Aldh1a1	TAGCAGCAGGAGTCTTCAC	ACAGCCAAATAGCAGTTCAC
Aldh1a3	CGGTCTTCTCAGATGTTACG	GAGTCCATAGTCGGTGCTA
Bdh1	ATACTTCCTCTGTCATCAAC	CCACCAGTAGTAGTCCAT
Crym	GGTCCAGGCGTACAGTCA	GGTGCGGTTCCACATTCTC
Scd3	GTTGCCACTTTACTGAGATACGC	GAAGCCCTCGCCCATACTT
Gldc	TCTGGAGGTCGTATATTG	GGTCTGGTAGTTGAGTAA
Mpc1	TTCGCCCTCTGTTGCTATT	GCCGCTTACTCATCTCGTA
Mpc2	TGGATAAAGTGGAGTTGTTG	TAAACCCTGTAGCCATCAA
Aco2	ATCTCTAACAACCTGCTCAT	CATAGTTCTCATCTCCAATCAC

Idh1	AGGAGGTTCTGTGGTGGAGAT	CCTGGTCATTGGTGGCATCA
Idh2	AACACCGACGAGTCCATT	GTCAGTCTTATAGTGCTTGTC<br>AA
Dlst	GGTCTCGTGGTTCCTGTCAT	CTCCTCCGTTGCTGATGGT
Sdha	GACATCAAGACTGGCAAGGTTA	GAGCGGATAGCAGGAGGTA
